# Intercalation‐Induced Phase Transitions in Ferroelectric α‐In_2_Se_3_

**DOI:** 10.1002/advs.202513712

**Published:** 2025-12-12

**Authors:** Xin He, Zhihao Gong, Tao Wang, Baoyu Wang, Chen Liu, Ding Wang, Yinchang Ma, Pu Feng, Chenhui Zhang, Weijin Hu, Kai Liu, Hua Wang, Xixiang Zhang

**Affiliations:** ^1^ Center for Quantum Matter School of Physics Zhejiang University Hangzhou 310058 China; ^2^ ZJU‐Hangzhou Global Scientific and Technological Innovation Center College of Integrated Circuits Zhejiang University Hangzhou 311215 China; ^3^ Academy of Interdisciplinary Studies on Intelligent Molecules Tianjin Key Laboratory of Structure and Performance for Functional Molecules College of Chemistry Tianjin Normal University Tianjin 300387 China; ^4^ Physical Science and Engineering Division King Abdullah University of Science and Technology Thuwal 23955‐6900 Saudi Arabia; ^5^ Shenyang National Laboratory for Materials Science Institute of Metal Research Chinese Academy of Sciences Shenyang 110016 China; ^6^ School of Materials Science and Engineering University of Science and Technology of China Shenyang 110016 China; ^7^ Physics Department Georgetown University Washington, DC 20057 USA

**Keywords:** ferroelectric α‐In_2_Se_3_, lithium intercalation, semiconductor–metal phase transitions

## Abstract

Specific ions can be intercalated into functional materials using the electrolyte gating technique, which has been widely used to regulate channel conductance in transistors and develop low‐power neuromorphic devices. However, in these devices, fundamental exploration of ion intercalation‐induced structural phase transitions remains largely overlooked and rarely explored. Here, the lithium‐based electrolyte gating technique is used to probe the collective interactions between ions, lattices, and electrons in a van der Waals ferroelectric semiconductor α‐In_2_Se_3_. Using a polymer electrolyte as the lithium‐ion reservoir and α‐In_2_Se_3_ as the channel material, the intercalated lithium concentration via a gate electric field is modulated. This manipulation drives a phase transition in α‐In_2_Se_3_ from a ferroelectric semiconductor to a dirty metal and finally to a metal, accompanied by a structural transformation. Concurrently, with enhanced intercalation, the ferroelectric hysteresis window progressively narrows and eventually disappears, indicating the evolution from switchable to non‐switchable polarization. This study represents a promising platform for the artificial construction of correlated material systems, enabling a systematic investigation into the interaction of ferroelectricity and electronic conduction using ion intercalation.

## Graphical abstract

Using the electrolyte gating technique, the van der Waals ferroelectric semiconductor α‐In_2_Se_3_ undergoes a series of transitions from a ferroelectric semiconductor to a dirty metal and finally to a metal, accompanied by a structural transformation. Concurrently, the ferroelectric hysteresis window progressively narrows and eventually disappears with enhanced lithium intercalation, indicating the evolution from switchable to non‐switchable polarization.

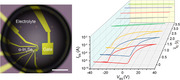

## 1 Introduction

In a metal‐oxide‐semiconductor field‐effect transistor (FET), the channel conductance is modulated by electrostatic depletion or accumulation of mobile carriers (i.e., electrons or holes) via a gate voltage. This architecture, with an oxide dielectric layer and a silicon channel, has brought many revolutionary technologies to the modern electronics industry.^[^ ^1^, ^2^, ^3^ ^]^ However, due to the relatively low dielectric constant of oxide materials and the normally weak gate electric field, the modulation range of channel carrier density remains small, which restricts the exploration of basic physics and technological applications under extreme conditions.

The electrolyte gating technique has recently attained growing interest for fundamental studies and device use.^[^ ^4^, ^5^, ^6^, ^7^, ^8^, ^9^ ^]^ This technique is able to tune carrier density over a wide range in FET architecture with a typical electrolyte instead of a conventional dielectric oxide.^[^ ^9^ ^]^ The electrolyte acts as an ion reservoir containing plenty of mobile cations and anions. Driven by electrostatic or electrochemical effects, these ions can aggregate at the channel surface or intercalate into the channel under a gate electric field, enabling the manipulation of many novel physical properties.^[^ ^8^, ^9^ ^]^ When large‐radius ions, e.g., various organic ions, are electrically pushed onto the channel, the electrostatic effect produces a ≈1 nm‐thick electric double layer at the electrolyte‐channel interface, inducing high‐density carriers of up to 10^14^ cm^−2^ over the film's top surface.^[^ ^8^, ^9^ ^]^ This electrostatic electrolyte gating is applicable to ultrathin nanoscale films without altering their structures, but is inappropriate for thick films due to electrostatic screening.^[^ ^10^ ^]^ By contrast, in thick films or bulk crystals, the electrochemical effect can promote ions (e.g., H^+^ and Li^+^ ions) entirely intercalating into the material interior, and generate both charge transfer (i.e., conductivity increase) and chemical bonds forming/breaking.^[^ ^9^ ^]^

Ion intercalation has been mostly used in chemical synthesis^[^ ^11^, ^12^ ^]^, liquid exfoliation^[^ ^13^, ^14^ ^]^, and energy harvesting.^[^ ^15^, ^16^ ^]^ Particularly, this technique has shown compelling prospects for low‐power neuromorphic computing devices.^[^ ^4^, ^17^, ^18^, ^19^, ^20^, ^21^, ^22^, ^23^, ^24^, ^25^, ^26^, ^27^, ^28^ ^]^ In a prior study^[^ ^29^ ^]^, we have reported on the creation of phase transitions using a back‐gate protonation approach, but the achieved carrier density was relatively small (≈ 6 × 10^12^/cm^2^) and insufficient to access a metal phase or correlated systems with rich physics. To address this issue, here we leverage top‐gate lithium intercalation due to the abundant ion sources in the electrolyte and gapless contact with the channel material. Other intercalants, notably sodium and potassium ions, were excluded due to concerns that their large sizes could introduce extensive defects in the host structures. Moreover, we select ferroelectric α‐In_2_Se_3_ semiconductor as the material of the transistor channel because α‐In_2_Se_3_ crystal can undergo phase transformations upon applying an energy‐sufficient stimulus^[^ ^30^, ^31^ ^]^, and the tunable carrier density in the channel can quickly reach that of a metallic phase. Besides, α‐In_2_Se_3_ crystal can retain ferroelectric polarization even down to a monolayer^[^ ^32^ ^]^, and has a moderate bandgap (1.4–1.9 eV).^[^ ^33^ ^]^

By employing a top‐electrolyte‐gating geometry with the ferroelectric semiconductor α‐In_2_Se_3_ as the channel material, we achieve a high carrier density up to 5.79 × 10^13^/cm^2^. This gate‐modulated lithium intercalation drives a phase transition in α‐In_2_Se_3_ from a semiconductor to a metal, which is accompanied by a structural transformation. The broadly gate‐tunable carrier concentration reveals collective interactions among polar ordering, structural deformation, and ion injection. Our results establish ion intercalation as a robust approach for the on‐device manipulation of phase transitions and the design of correlated material systems.

## 2 Results and Discussion

### 2.1 The Approach to Triggering Phase Transition

**Figure 1:**
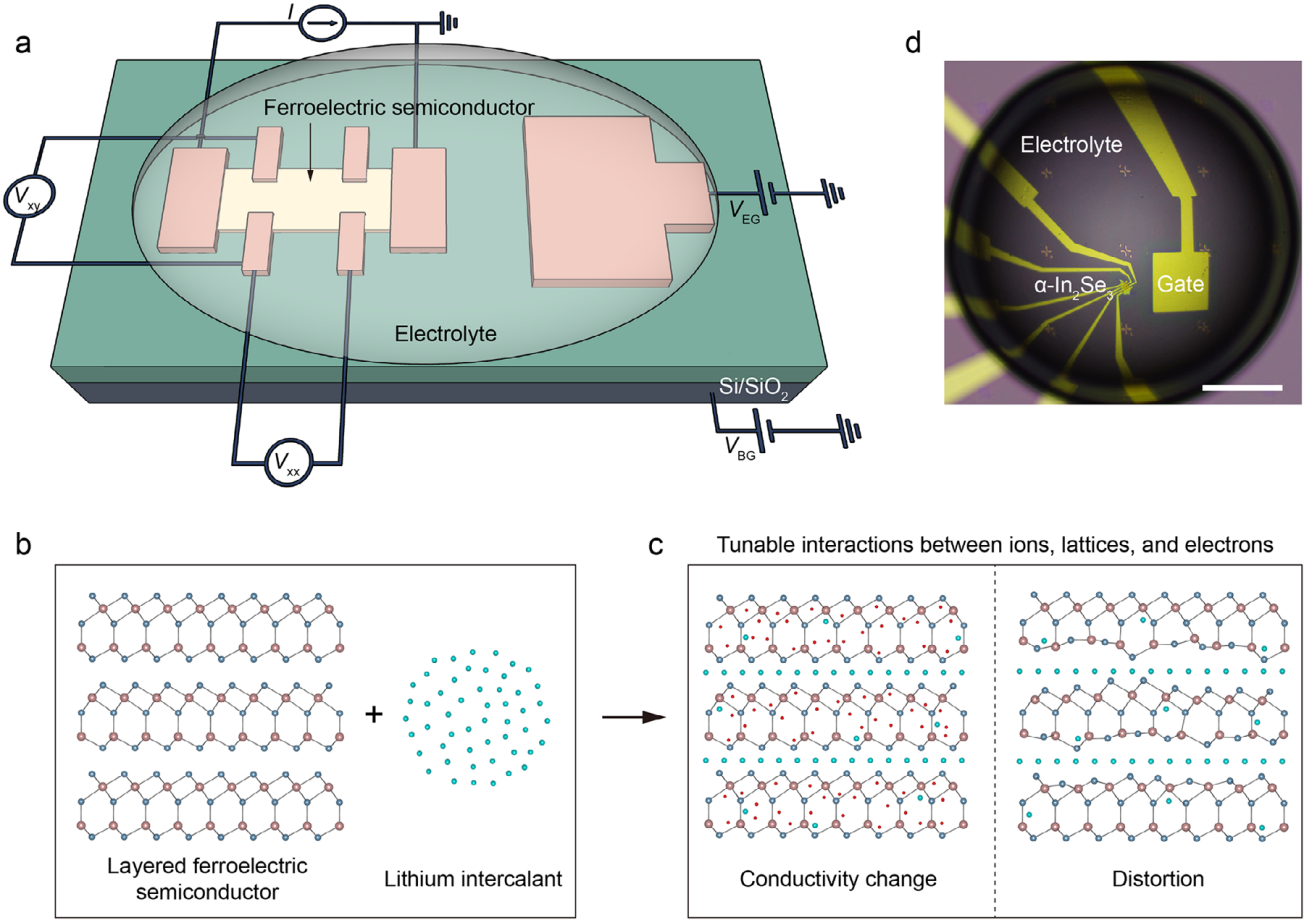
An approach for inducing on‐device phase transitions. a) Schematic diagram of our on‐device platform that implements intercalation processes. Narrow‐bandgap semiconducting ferroelectrics, other than insulating ferroelectrics, act as channel material, while the top electrolyte and gate electrode on the right panel serve for electrolyte gating. b, c) Illustration of the intercalation processes over the device channel. A few typical effects, e.g., conductivity change and structural distortion, are involved upon intercalation. Intercalation provides a device platform for studying the collective interactions between ions, lattices, and electrons. Red dots in (c) indicate itinerant electrons. d) Optical image of a typical device with an α‐In_2_Se_3_ channel covered by a LiClO_4_‐PEO electrolyte. Scale bar: 100 µm.

We propose a strategy to implement the intercalation phase transition on a device level. **Figure** **1**a presents the schematic structure of a lithium‐based electrolyte‐gated device, in which the top gate comprises a polymer electrolyte, and the preparation method can be found in a previous study.^[^ ^34^ ^]^ An electrolyte‐gate voltage (*V*_EG_) is used to inject Li ions into or extract Li ions from the channel to tune crystal phases, while a back‐gate voltage (*V*_BG_) is employed to switch the channel polarization for reading out the dynamics of structural phase transition. The longitudinal and transverse voltages (*V*_xx_ and *V*_xy_) are used to calculate the resistance and carrier density of the channel, respectively.

Schematics in Figure 1b,c indicate that lithium intercalation can produce a few fundamental effects in a layered material, e.g., carrier doping and lattice distortion. Nevertheless, the latter effect has been largely overlooked in most electrolyte‐gating transistors.^[^ ^18^, ^19^, ^20^, ^21^, ^22^ ^]^ In principle, these two effects can be manifested by the change in channel conductivity, thickness, or structural deformation. Figure 1d shows an optical image of our actual transistor device, whose fabrication details are presented in the Experimental Section. The α‐In_2_Se_3_ ferroelectricity is confirmed by spontaneous ferroelectric domains and switching loops in Figure S1a‐c (Supporting Information), consistent with previous works.^[^ ^35^, ^36^, ^37^ ^]^ Furthermore, we performed first‐principles calculations (Figure S2, Supporting Information) to shed light on the theoretical structural change with heavy lithium intercalation, i.e., a new compound of α‐In_2_Se_3_Li*_x_*. Specifically, a single Li atom is placed in a unit cell of pristine α‐In_2_Se_3_, which equals a doping concentration of ≈ 3.8 × 10^21^/cm^3^ and is much greater than the experimental cases that we can achieve. We find that two types of α‐In_2_Se_3_Li*_x_* crystal structures are energetically favorable, formed by intralayer (Figure S2a, Supporting Information) and interlayer (Figure S2b, Supporting Information) intercalation, respectively, which demonstrates the interaction between lithium ions and α‐In_2_Se_3_ lattices, and coincides with the proton intercalation in our previous study.^[^ ^29^ ^]^ In addition, for these two types of intercalation scenarios, the calculated charge transfer between Li ions and bilayer ɑ‐In_2_Se_3_ is shown in Figure S3a,b (Supporting Information), indicating that both new compounds can stabilize. Particularly, the interlayer‐intercalated α‐In_2_Se_3_Li*_x_* compounds are more stable since the calculated binding energy is ≈100 meV lower than that of intralayer intercalation. Therefore, interlayer intercalation is energetically preferred during the intercalation process, and the intercalation compounds are mainly interlayer‐intercalated α‐In_2_Se_3_Li_x_.

### 2.2 Probing Intercalation‐Induced Phase Transitions

**Figure 2:**
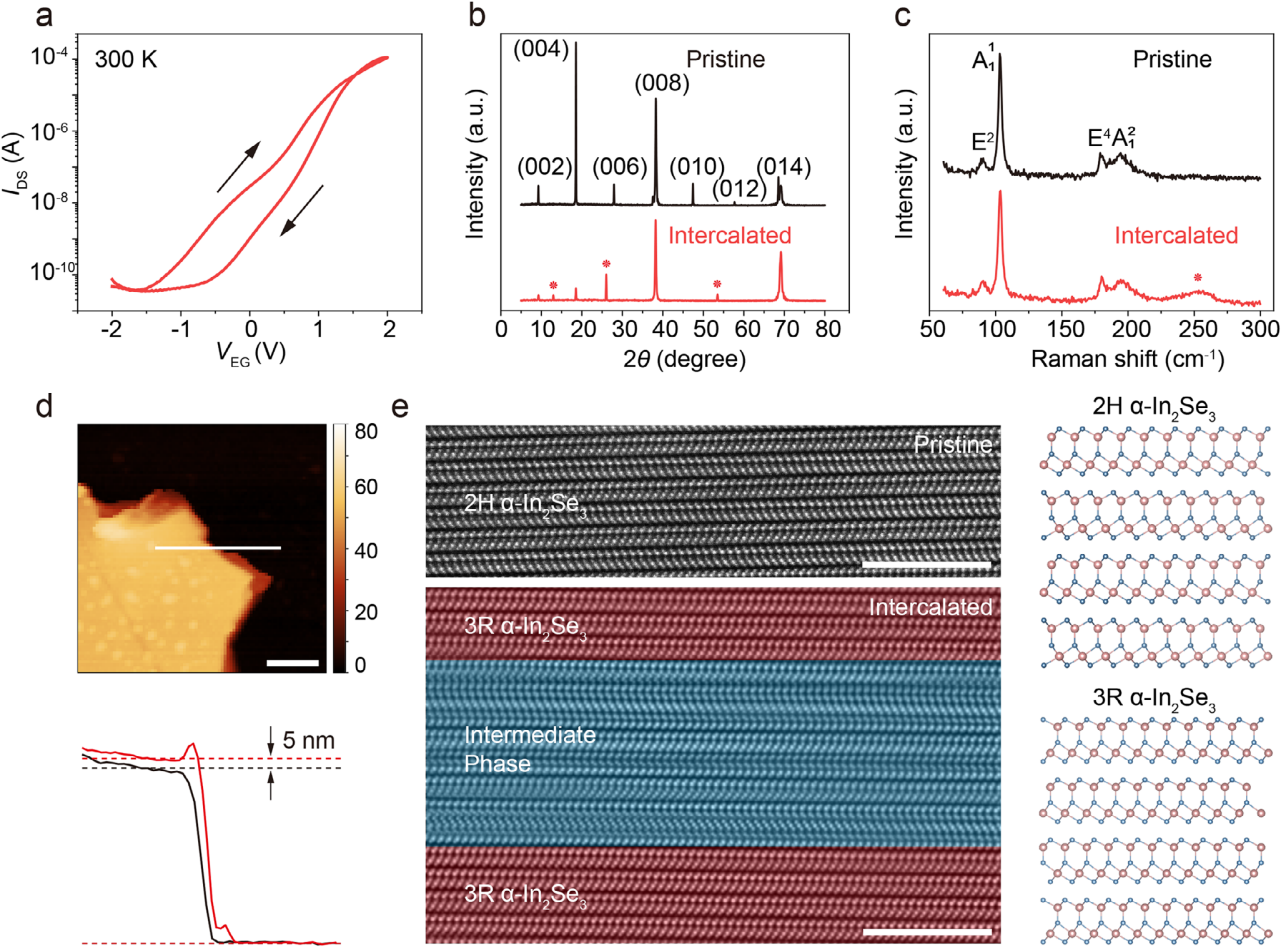
Intercalated structural change accompanied by a conductivity increase. a) Hysteresis transfer curves of a typical *α*‐In_2_Se_3_ device under electrolyte gating. The arrows dictate sweeping directions. b) XRD patterns of pristine and Li‐intercalated *α*‐In_2_Se_3_ flakes. Red stars denote the emerging peaks. c) Raman spectra before and after Li‐intercalation. The red star indicates a new vibrational peak. d) AFM image of an α‐In_2_Se_3_ flake and the corresponding thickness profiles for the pristine and intercalated states, which were extracted along the white lines. Scale bar: 200 nm. e) STEM images for pristine and Li‐intercalated samples, demonstrating the phase transition from a 2H *α*‐In_2_Se_3_ phase to a mixed In_2_Se_3_Li_x_ phase consisting of intermediate phases and a 3R *α*‐In_2_Se_3_ phase. Scale bars: 5 nm.

To explore the phase transitions and ion intercalation in our devices, top‐electrolyte‐gate transfer curves were first acquired. Figure 2a shows a bidirectional *V*_EG_‐bias sweeping over a narrow range (±2 V) at 300 K, rather than at a low temperature. This is because low temperatures can freeze and even impede lithium injection. The transfer curve (*I*_DS_ – *V*_EG_) shows an apparent n‐type conducting behavior and an on–off ratio of up to 10^6^, which demonstrates the significant doping effect resulting from lithium intercalation as indicated in Figure 1c. The chemical reaction of the Li‐ion intercalation occurs during the forward *V*_EG_ sweep (α‐In_2_Se_3_ + *x*Li^+^ + *x*e^−^ → α‐In_2_Se_3_Li*_x_*), and the deintercalation of Li ions takes place during the backward *V*_EG_ sweep (α‐In_2_Se_3_Li*_x_* → α‐In_2_Se_3_ + *x*Li^+^ + *x*e^−^), indicating the interaction between lithium ions and electrons. Furthermore, a clockwise hysteresis loop is observed, as shown in **Figure** **2**a, which primarily originates from different α‐In_2_Se_3_Li_x_ phases and ion migration, similar to that of our previous study.^[^ ^29^ ^]^ On the other hand, to examine the polarization switching at the channel, the *V*_BG_ bias was scanned on the same device to obtain back‐gate transfer curves (*I*_DS_−*V*_BG_), as shown in Figure S4 (Supporting Information). Following published works^[^ ^37^, ^38^, ^39^ ^]^, upon the sweeping of the large back‐gate *V*_BG_, the hysteresis shall result from the intrinsic polarization reversal of the α‐In_2_Se_3_Li*_x_* channel. An increase in the maximum scanning voltage *V*_BG_ can broaden hysteretic windows and switch more ferroelectric domains.^[^ ^39^, ^40^ ^]^ Specifically, a sufficiently positive *V*_BG_ can reverse domains pointing upward, whereas a sufficiently negative *V*_BG_ compels domains pointing downward. Moreover, all the back‐gate hysteresis loops also exhibit a clockwise behavior owing to the semiconducting nature of ferroelectrics, which agrees with previous studies.^[^ ^37^, ^38^, ^39^, ^40^ ^]^

Besides the marked increase in conductivity, intercalation‐induced structural distortion or transformation is corroborated by other characterization techniques. Figure 2b displays X‐ray diffraction (XRD) patterns of pristine and Li‐intercalated α‐In_2_Se_3_ flakes. Compared with the XRD pattern of pristine α‐In_2_Se_3_ crystal, three original peaks at 27.958°, 47.441°, and 57.735° vanish while three new peaks at 12.960°, 26.040°, and 53.503° appear, implying the emergence of new phases in the intercalated compound α‐In_2_Se_3_Li_x_. For Raman spectra with lithium intercalation (Figure 2c; Figure S5, Supporting Information), a new broad vibrational peak can be observed at around 254 cm^−1^, which is consistent with previous reports and ascribed to Se_8_ rings.^[^ ^41^, ^42^ ^]^ The Se_8_ ring molecules may be formed by the separate Se in the intercalated compound with coexisting phases, whose composition has changed during the lithium intercalation. As mentioned above, the doping concentration in the experiment is significantly lower than that applied in the calculation. As a result, the actual lattice deformation is much weaker than the theoretical prediction and thus may be insufficient to induce the expected variation in the XRD pattern and Raman spectra. Nevertheless, the emergence of new peaks in both the XRD and Raman spectra still confirms successful intercalation. These new features likely correspond to a mixed phase of 3R α‐In_2_Se_3_ and intermediate phases, which will be discussed later. Moreover, atomic force microscopy (AFM) measurement provides direct evidence for variations in channel thickness before and after Li ion injections. The intercalated thickness was found to increase by ≈9.4% (Figure 2d), which could be attributed to the formation of new compounds and their lattice expansion.

To capture the atomic structure transition, scanning transmission electron microscopy (STEM) images for pristine and Li‐intercalated α‐In_2_Se_3_ (i.e., α‐In_2_Se_3_Li*_x_*) were obtained as well. As shown in Figure 2e, pristine α‐In_2_Se_3_ exhibits a typical hexagonal (2H) structure.^[^ ^43^ ^]^ During intercalation, the α‐In_2_Se_3_ crystal undergoes deformation and is driven to a high‐energy state with a high‐carrier density (Figure S2, Supporting Information). But after intercalation, the compound relaxes to a low‐energy state, wherein an intermediate phase and a rhombohedral (3R) phase of α‐In_2_Se_3_ form and coexist (Figure 2e). In the intermediate phase region as highlighted by blue, the atomic layers experience intralayer splitting and interlayer reconstruction, constituting a mixed phase (Figure 2e). These results suggest that the experimentally realized α‐In_2_Se_3_Li*_x_* compounds indeed encompass 3R α‐In_2_Se_3_ and mixed intermediate phases.

### 2.3 Semiconducting‐to‐Metallic Phase Transition

**Figure 3:**
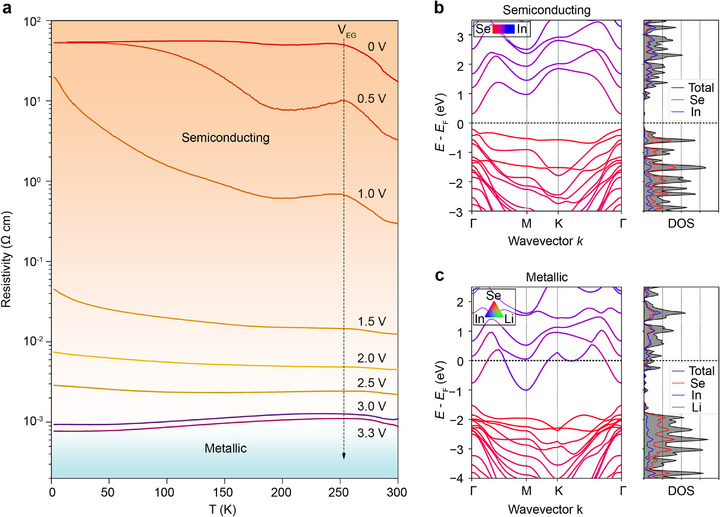
Semiconducting‐to‐metallic phase transition by on‐device lithium intercalation. a) Temperature‐dependent resistivity of a 42‐nm‐thick α‐In_2_Se_3_Li_x_ flake under various *V*_EG_, exhibiting a semiconductor‐to‐metal phase transition with increasing *V*_EG_. Calculated band structure and density of states (DOS) for b) initial bilayer ɑ‐In_2_Se_3_ and c) metallic bilayer ɑ‐In_2_Se_3_ with interlayer lithium intercalation.

**Figure 4:**
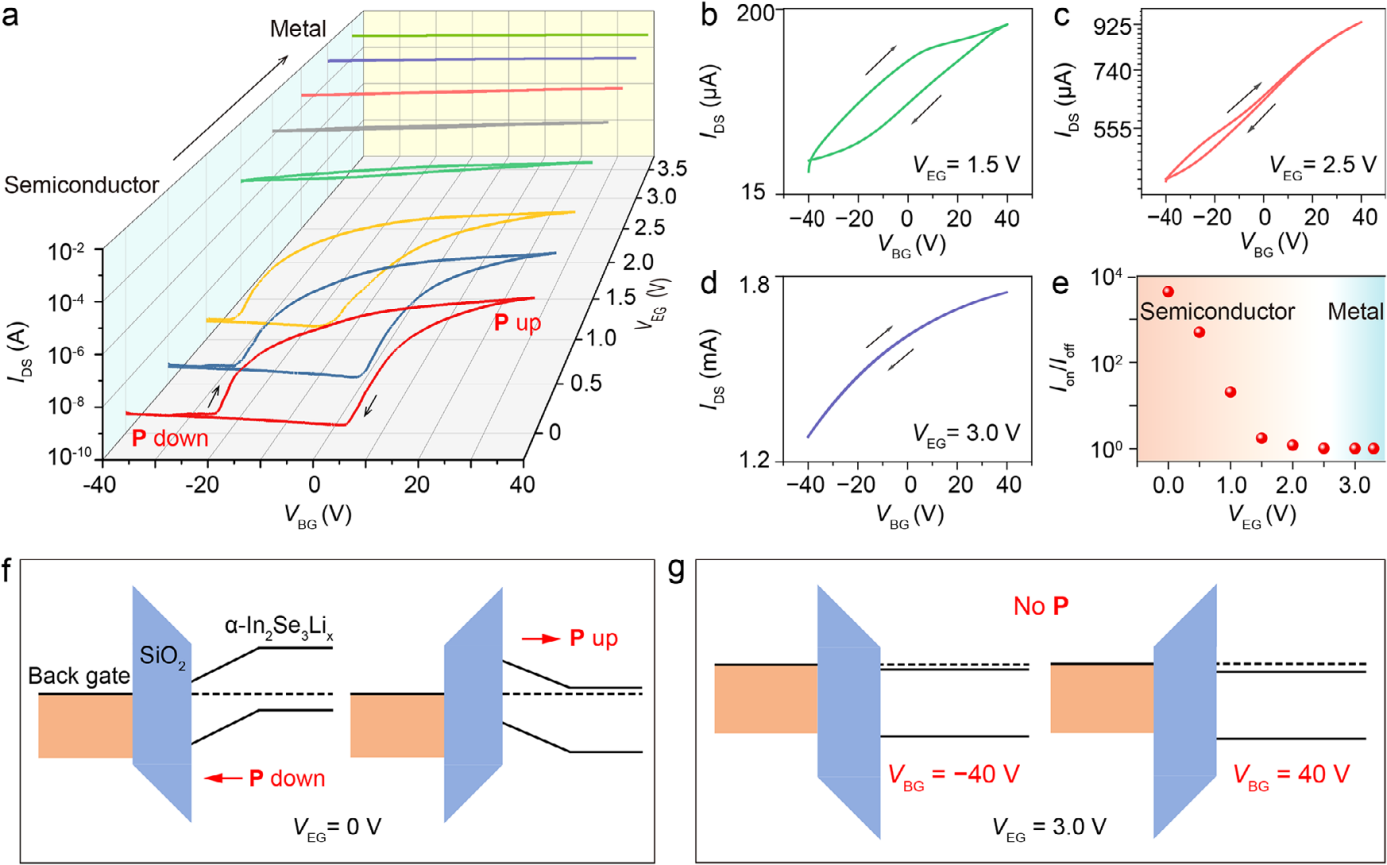
Dynamic phase transition to a metallic state. a) Evolution of the transfer curves with bidirectional sweeping *V*_BG_ and various *V*_EG_ at 2.5 K, indicating the transition from a ferroelectric semiconductor to a dirty metal, and then to a metal (*V*_DS_ is 1.0 V). b–d) Selected hysteresis transfer curves at *V*_EG_ = 1.5, 2.5, and 3.0 V. Data is extracted from (a). e) Memory on/off ratio vs *V*_EG_ read at *V*_BG_ = 5.0 V for showing hysteresis variation. f) Band diagrams of the α‐In_2_Se_3_Li_x_ device at *V*_EG_ = 0 V, elucidating the origin of switchable hysteresis in (a). g) Band diagrams of the α‐In_2_Se_3_Li_x_ device at *V*_EG_ = 3.0 V, elucidating the vanishment of switchable hysteresis in (d).

To demonstrate conductivity‐dependent ferroelectricity and related phase‐transition dynamics, resistivity–temperature (*R*–*T*) curves (**Figure** **3**) and transfer curves (Figure 4) were obtained at each cooling cycle with the same device. During the measurement, once a stable lithium‐intercalated state was formed at 300 K, the sample was cooled while its resistivity was monitored in situ using a four‐point configuration, as shown in Figure 3; when the sample temperature reached 2.5 K, hysteresis transfer curves were acquired by sweeping *V*_BG_ in a transistor configuration, as illustrated in Figure 4. It is worth noting that the intercalation is performed at 300 K rather than at low temperatures, because lithium ions become less mobile and even frozen at low temperatures. Figure 3a shows the low‐temperature *R*‐*T* transport properties of the device with respect to different *V*_EG_. As shown by the dashed arrow, with increasing top‐gate *V*_EG,_ the channel resistivity markedly decreases, indicating efficient lithium intercalation. Under low voltages (e.g., *V*_EG_ = 0 and 0.5 V), with decreasing temperature, the channel resistivity increases and eventually reaches 53.7 Ω·cm at 2.5 K. For these cases, the variation of *R*–*T* curves is steep, suggesting remarkable semiconducting behaviors. However, when *V*_EG_ increases to 2.5 V, the channel resistivity initially decreases with cooling from ≈250 to ≈150 K but then increases upon further cooling to 2.5 K, demonstrating a dirty metal behavior. Here, a dirty metal state corresponds to a state that displays semiconducting behavior in the low temperature range, while metallic behavior in the high temperature range. Unexpectedly, after *V*_EG_ is set to 3.0 or 3.3 V, the channel resistivity evidently decreases with decreasing temperature despite the bump at ≈250 K, which suggests the transition to a metal phase. Therefore, we conclude that an unambiguous semiconductor‐to‐metal transition occurs at around *V*_EG_ = 3.0 V.

To quantitatively analyze the electronic phase transitions, we detected both carrier density and mobility with respect to *V*_EG_ by using magneto‐transport measurements at 2.5 K (Figure S6a,b, Supporting Information). The carrier density and mobility of α‐In_2_Se_3_Li*_x_* compounds were extracted, and found to increase monotonically with increasing *V*_EG_, reaching their highest values (5.79 × 10^13^/*cm* ^2^  and 505 *cm* ^2^/*Vs*) at *V*_EG_ ═ 3.3 V (Figure S6c, Supporting Information). It is worth noting that these two variables could not be obtained below *V*_EG_ ═1.5 V since the small Hall signal was overwhelmed by the large magnetoresistance background. We find that the α‐In_2_Se_3_Li*_x_* channel becomes metallic at *n* ═ 5.21 × 10^13^/cm^2^ (*V*_EG_ ═ 3.0 V), one order of magnitude larger than the phase transition concentration reported in a previous report^[^ ^38^ ^]^, which is due to the inhomogeneous carrier distribution in their α‐In_2_Se_3_ channel, and only the bottom layers of α‐In_2_Se_3_ become metallic. Such a high carrier density contributes to semiconductor–metal transitions and reveals that lithium intercalation can generate pronounced phase transitions than that of the traditional electrostatic back‐gating method.^[^ ^38^ ^]^ Additionally, we carried out first‐principles calculations on pristine bilayer α‐In_2_Se_3_ and intercalated flakes to provide insights into the phase transitions. Figure 3b,c and Figure S7 (Supporting Information) show that both the energy band structures and Fermi levels can be modified by lithium intercalation. With heavy lithium intercalation, the α‐In_2_Se_3_Li*_x_* compound can transform into a degenerate semiconductor and show metallic properties because the Fermi level is shifted into the conduction band (Figure 3c; Figure S7, Supporting Information). These theoretical results are consistent with the experimental conductance change shown in Figure 3a.

### 2.4 Dynamic Control of Ferroelectricity‐Induced Hysteresis

Phase transitions, particularly the dynamics of structural distortion or change, present huge challenges in terms of detection. We introduce the lithium intercalation method to trigger different phases and enable the macroscopic monitoring of dynamic phase transition by back‐gate sweeping. To study phase transitions and greatly reduce the influence of other factors (e.g., ion migration and charge trapping) on electrical hysteresis, cryogenic measurements as low as 2.5 K were conducted (**Figure** **4**a–d; Figure S8a–h, Supporting Information). Figure 4a shows a series of *V*_EG_‐dependent back‐gate transfer curves with hysteresis for the same device whose R‐T curves are shown in Figure 3a. Figure 4b–d display three representative transfer curves, which correspond to the moderate (*V*_EG_ = 1.5, 2.5 V) and enhanced (*V*_EG_ = 3.0 V)‐intercalation states, respectively. All the transfer curves display a clockwise behavior^[^ ^37^ ^]^ and the drain‐source current obviously increases with raised *V*_EG_, manifesting enhanced electron concentrations inside the α‐In_2_Se_3_Li*_x_* channel. Since charge trapping is greatly suppressed at a liquid‐helium temperature, the observed hysteresis loops primarily originate from the α‐In_2_Se_3_Li*_x_* polarization switching.^[^ ^37^, ^38^ ^]^ Moreover, the hysteresis in these transfer curves weakens with increasing *V*_EG_ and finally vanishes at *V*_EG_ = 3.0 V.

Specifically, upon *V*_EG_ ═ 0 V (Figure 4a; Figure S8a, Supporting Information), the hysteresis loop saturates when *V*_BG_ reaches 40 V, and shows a large on/off ratio. As *V*_EG_ is raised to 1.0 V (Figure  4a; Figure  S8c, Supporting Information), a great amount of Li ions and electrons are injected into the α‐In_2_Se_3_Li*_x_* compound, and the electrical loop shifts toward the top‐left direction with the reduction of *V*_BG_ threshold voltage. At *V*_EG_ ═1.5 V (Figure  4b; Figure  S8d, Supporting Information), significant shrinking of the hysteresis loop quickly suppresses the on/off ratio to below 10. Such a behavior could be explained by the intercalation‐induced perturbation effect in long‐range dipole–dipole interactions and enhanced electrostatic screening effects, highlighting the interaction between electrons and α‐In_2_Se_3_ lattices. This behavior aligns well with the abrupt drop of the channel resistivity at *V*_EG_ ═ 1.5 V in Figure 3a. It is emphasized that the great number of intercalated ions and injected electrons across the channel could destroy the balance between long‐range Coulomb forces and short‐range electron repulsion, exerting a strong influence on lattice instability and structural phase transition.^[^ ^44^ ^]^

As *V*_EG_ is further raised to 2.0 V (Figure S8e, Supporting Information) or 2.5 V (Figure 4c; Figure S8f, Supporting Information), the electrical hysteresis loop continues to shrink but is still observable, indicating that the polar order can survive, at least, with a carrier density up to 5.20 × 10^13^/*cm* ^2^ (*V*_EG_ = 2.5 V). When the sample completely transforms to a metallic phase under *V*_EG_ = 3.0 V (Figure 4d; Figure S8g, Supporting Information) or 3.3 V (Figure S8h, Supporting Information), the hysteresis completely disappears. This behavior suggests the complete transition of α‐In_2_Se_3_Li*_x_* from a ferroelectric semiconductor to a metal. We note that, in Figure 4e, the phase transition dynamics can be manifested by the relationship between the memory on–off ratio (*I*_ON_/*I*_OFF_ at *V*_BG_ ═ 5.0 V) and *V*_EG_. For instance, by increasing *V*_EG_, the ratio is reduced from 4.3 × 10^3^ (*V*_EG_ = 0 V) to 1.7 (*V*_EG_ = 1.5 V). The ratio approaches almost 1 once *V*_EG_ is further increased to 3.0 V, which demonstrates tunable intercalation phase transitions. Additionally, in Figure 4f, we plot band diagrams for the α‐In_2_Se_3_Li*_x_* transistors with switchable polarization (*V*_EG_ = 0 V) in both high‐ and low‐resistance states, as marked in Figure 4a. While the band diagrams for the α‐In_2_Se_3_Li*_x_* transistors without switchable polarization are shown in Figure 4g (*V*_EG_ = 3.0 V). Note that the energy bands are simplified by ignoring the semiconducting nature‐induced band bending^[^ ^37^ ^]^ and lattice‐expansion and ‐distortion induced band modification. For other lithium intercalation levels, a more detailed analysis of the band diagrams can be found in Figure S9 and Note S1 (Supporting Information).

## 3 Conclusion

Herein, we have demonstrated a lithium‐intercalation‐induced phase transition on a transistor level. A polymer electrolyte gate was used to intercalate Li ions into the multilayer ferroelectric semiconductor α‐In_2_Se_3_ to tune the phase transitions. By measuring *R*–*T* and transfer curves, the dynamic phase transition of α‐In_2_Se_3_ from a ferroelectric semiconductor to a dirty metal and then to a metal has been demonstrated, which is accompanied by the evolution of electric polarization from switchable to non‐switchable, as well as a structural transformation. This study offers insight into the relationship between ferroelectricity and electronic conduction through intercalation‐induced phase transitions.

Our work provides a pragmatic approach to manipulating dynamic properties between ion, lattice, and electron over a wide conductivity range. This intercalation approach could help explore unprecedentedly rich physics related to the collective interactions of lattice distortion, charges, and spins.

## 4 Experimental Section

### Device Fabrication

α‐In_2_Se_3_ flakes were exfoliated onto Si/SiO_2_ (500 µm/300 nm) substrates from bulk α‐In_2_Se_3_ crystal (2D Semiconductors Inc.). After flakes with the proper shapes and thicknesses were identified using an optical microscope (Carl Zeiss Imager.A2 Vario with AxioCam HRc), Hall bars and electrolyte gate electrodes were fabricated using e‐beam lithography and e‐beam deposition (Ti/Au: 10 nm/70 nm). Afterward, a small drop of polymer electrolyte^[^ ^34^ ^]^ was dripped onto the devices and then solidified. Following a previously reported method^[^ ^34^ ^]^, the electrolyte was prepared by mixing LiClO_4_ (0.3 g) and polyethylene oxide powder (1 g, M_w_ = 100 000) in 15‐mL anhydrous methanol. Subsequently, the resulting solution was sealed and stirred at 50 °C for 12 h.

### Characterizations

The devices were delivered to a Quantum Design physical property measurement system (PPMS) after fabrication. During the electrical measurements, alternating currents and gate voltages were applied using a Keithley 6221 current source and a Keithley 2636B voltage source, respectively, and an SR830 lock‐in amplifier was used to measure the voltages of the Hall bars. A Keithley 4200‐SCS semiconductor characterization system was employed to characterize the electronic properties of the ɑ‐In_2_Se_3_Li_x_ transistors. At the beginning of each cooling cycle, a *V*_EG_ was applied and held at 300 K for half an hour to obtain uniform lithium intercalation. The X‐ray diffraction (XRD) patterns were collected on a Bruker D8 Discover X‐ray diffractometer. The Raman spectra were obtained on a Horiba LabRAM Odyssey Raman microscope. The thicknesses of the α‐In_2_Se_3_ flakes were confirmed by AFM (Asylum Research MFP‐3D or Bruker Dimension XR). The ferroelectric properties of α‐In_2_Se_3_ were characterized in Asylum Research MFP‐3D. The cross‐sectional transmission electron microscopy (TEM) lamella of the device was fabricated by the focused ion beam (FIB) technique (Helios G4 UX, FEI). The atomic resolution high‐angle annular dark‐field (HAADF)‐scanning transmission electron microscopy (STEM) images were obtained using FEI Titan Themis Cubed G2 300 (Cs Probe) TEM at 300 kV.

### First‐Principles Calculations

The numerical results were obtained by performing Density Functional Theory (DFT)^[^ ^45^, ^46^ ^]^ calculations using the Vienna Ab initio Simulation Package (VASP) code.^[^ ^47^, ^48^ ^]^ To determine relaxed structures, total energies, and charge transfer, the generalized gradient approximation (GGA) functional with the Perdew–Burke–Ernzerhof (PBE) parameterization was employed in estimating electronic exchange‐correlation energies^[^ ^49^, ^50^, ^51^ ^]^ The dispersion correction energy proposed by Grimme's D3 approach was incorporated to effectively account for van der Waals interactions between ɑ‐In_2_Se_3_ layers.^[^ ^52^ ^]^ For the calculations of band structures and density of states (DOS), the Heyd–Scuseria–Ernzerhof screened hybrid functional (HSE06) was alternatively implemented.^[^ ^53^ ^]^ In terms of accuracy, a cutoff energy of 400 eV was set for the plane‐wave basis, and the Monkhorst‐Pack k‐point sampling was performed using a 12 × 12 × 1 grid. The obtained results were consistently converged using a convergence criterion of 0.02 eV Å^−1^ for residual forces during ion relaxation and 10^−6^ eV for energy differences between successive self‐consistent‐field (SCF) calculation steps.

A slab model of 2D α‐In_2_Se_3_ materials consisting of bilayer ɑ‐In_2_Se_3_ with a vacuum space of considerable thickness was employed to avoid the interactions between periodic images of slabs. To investigate both interlayer and intralayer lithium intercalations, a single Li atom was inserted into corresponding positions in the unit cell. Note that one Li atom per unit cell corresponds to a carrier density of ≈3.8 × 10^21^/cm^3^, which is an extreme case in calculations and much larger than what is typically achieved in experiments. For lower‐density doping, the Fermi level will shift down slightly, but this will not affect the conclusions. Given the possibility of the DFT relaxation process getting trapped at metastable states, a standard strategy was followed of utilizing multiple initial configurations to identify the optimal structure with the highest stability. Figure S3 (Supporting Information) illustrates the resulting structures with the lowest total energy for both interlayer and intralayer cases. In addition, the results of the charge transfer between Li and ɑ‐In_2_Se_3_ were plotted using the VESTA visualization software^[^ ^54^ ^]^, and corresponding effective values of total transfer charges for each ion were obtained by the Bader charge analysis.^[^ ^55^ ^]^

## Conflict of Interest

The authors declare no conflict of interest.

## Supporting information



Supporting Information

## Data Availability

The data that support the findings of this study are available from the corresponding author upon reasonable request.
