# Supplementary material for: Intercalation‐Induced Phase Transitions in Ferroelectric α‐In2Se3
Source: Adv Sci (Weinh). 2025 Dec 12;13(9):e13712. doi: 10.1002/advs.202513712 (PMC12903984; doi:10.1002/advs.202513712)
Supplement: Supplementary file 1 — Supporting Information [file ADVS-13-e13712-s001.docx]

Supporting Information for

**Intercalation-induced phase transitions in ferroelectric α-In_2_Se_3_**

*Xin He,^1,2#^ Zhihao Gong,^3#^ Tao Wang,^1,2#^ Baoyu Wang,^1,2^ Chen Liu,^4^ Ding Wang,^1,2^ Yinchang Ma,^4^* *Pu Feng,^1,2^ Chenhui Zhang,^4^ Weijin Hu,^5,6^ Kai Liu,^7^ Hua Wang,^1*^ and Xixiang Zhang^4*^*

^1^Center for Quantum Matter, School of Physics, Zhejiang University, Hangzhou 310058, China.

^2^ZJU-Hangzhou Global Scientific and Technological Innovation Center, College of Integrated Circuits, Zhejiang University, Hangzhou, 311215, China.

^3^Academy of Interdisciplinary Studies on Intelligent Molecules, Tianjin Key Laboratory of Structure and Performance for Functional Molecules, College of Chemistry, Tianjin Normal University, Tianjin 300387, China.

^4^Physical Science and Engineering Division, King Abdullah University of Science and Technology, Thuwal 23955-6900, Saudi Arabia.

^5^Shenyang National Laboratory for Materials Science, Institute of Metal Research, Chinese Academy of Sciences, Shenyang 110016, China.

^6^School of Materials Science and Engineering, University of Science and Technology of China, Shenyang 110016, China.

^7^Physics Department, Georgetown University, Washington, DC 20057, USA.

^#^These authors contributed equally

*Email: daodaohw@zju.edu.cn (H.W.); xixiang.zhang@kaust.edu.sa (X.Z.)


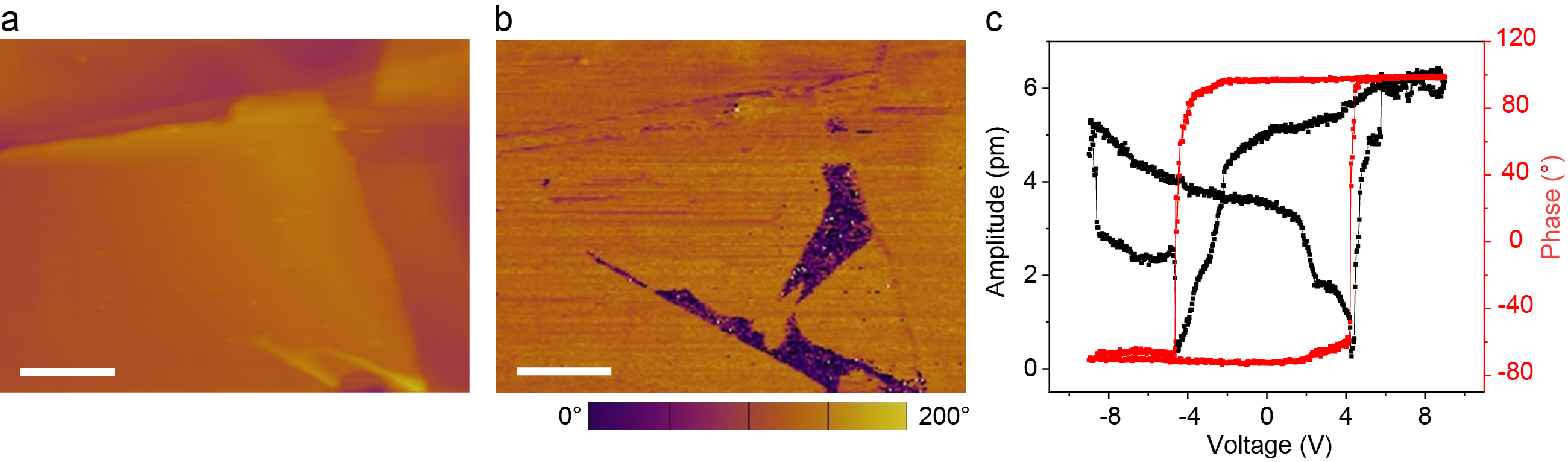


**Figure S1.** PFM topographic image a) and corresponding phase image b) of a typical α-In_2_Se_3_ flake with a thickness of ~150 nm. Scale bars: 1 µm. c) PFM amplitude and phase hysteresis loops of a typical α-In_2_Se_3_ flake.


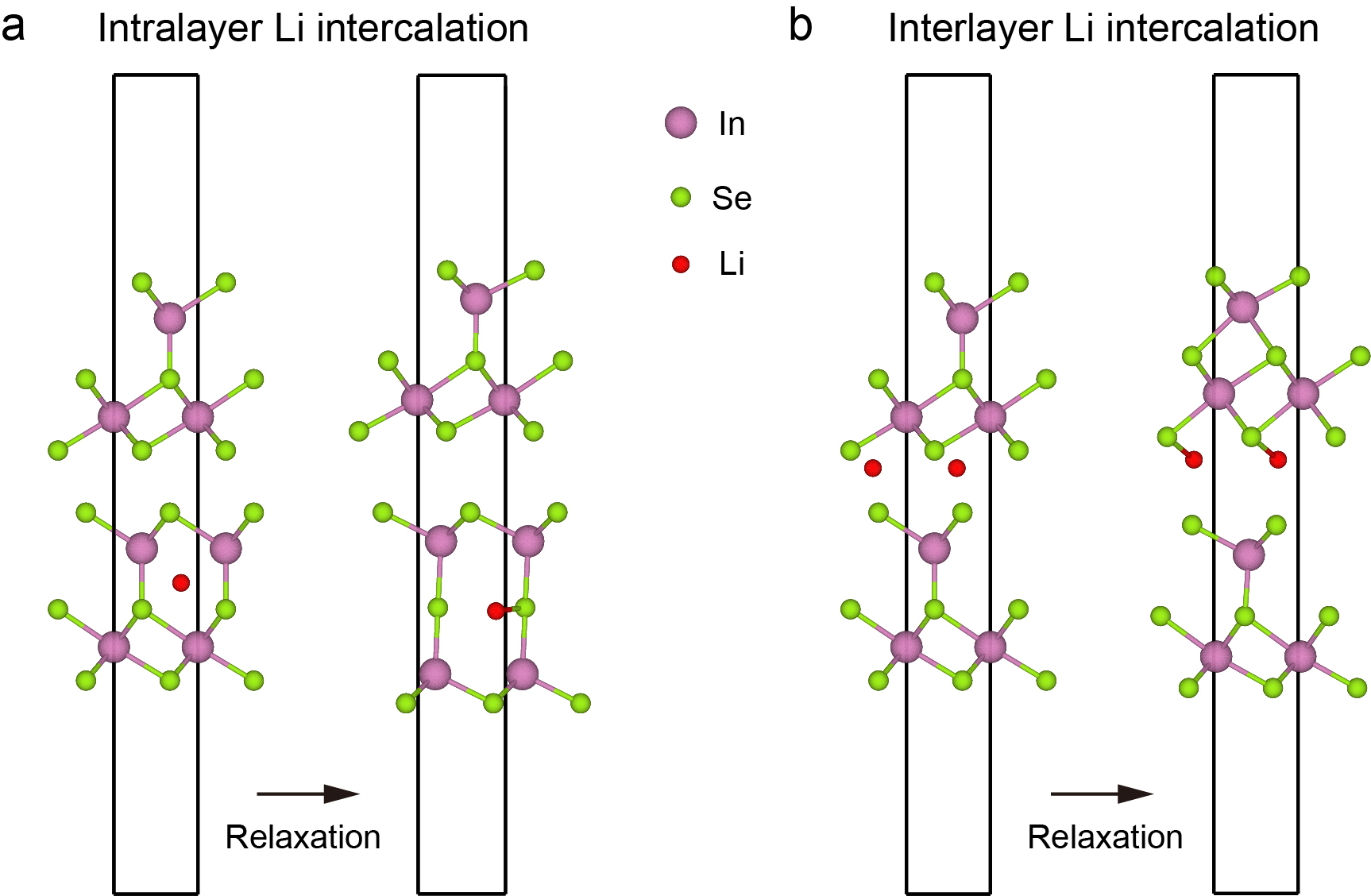


**Figure S2.** First-principles structural relaxation of α-In_2_Se_3_ with intralayer lithium intercalation a) and interlayer lithium intercalation b).


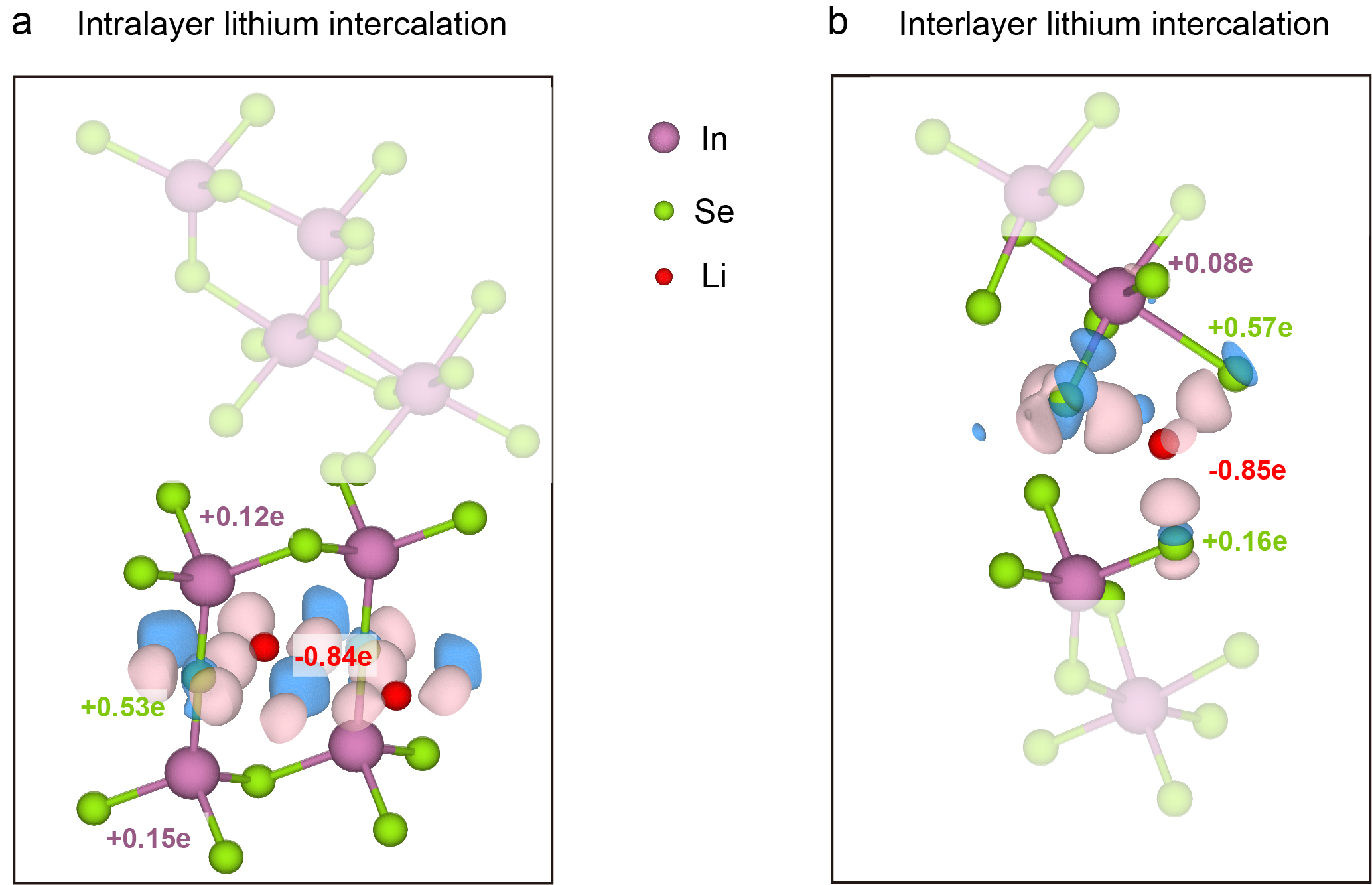


**Figure S3.** Charge transfer. Spatial distributions of charge transfer between Li ions and bilayer ɑ-In_2_Se_3_ in intralayer a) and interlayer b) lithium intercalation. Pink and blue isosurfaces represent positive and negative charge densities, respectively, with an isosurface level of$2.5\times{10}^{-3}e/{Bohr}^{3}$ . The amounts of Bader charges for Li, In, and Se ions are displayed in red, purple, and green colors, respectively. Major charge densities are found to be transferred from Li ions to neighboring Se ions, and minor charge densities are conveyed to the next-neighboring In ions. The results presented for both spatial distributions and Bader charges indicate that the bonds formed between Li ions and Se ions are fairly stable.


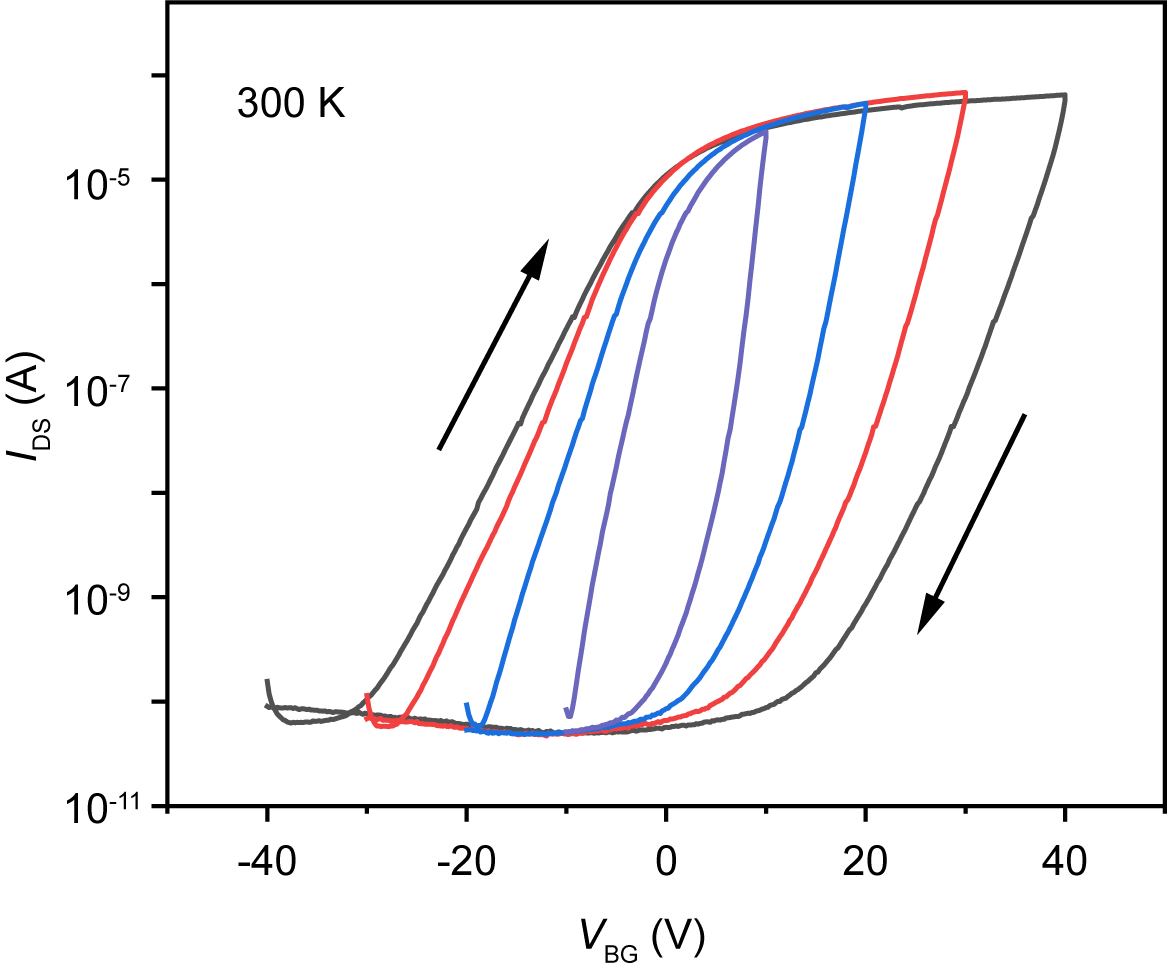


**Figure S4.** Hysteresis transfer curves via sweeping *V*_BG_ of different ranges at 300 K for an ɑ-In_2_Se_3_Li_x_ device. The transfer curves in Figure 2 and S4 were all obtained from the same ɑ-In_2_Se_3_Li_x_ device.


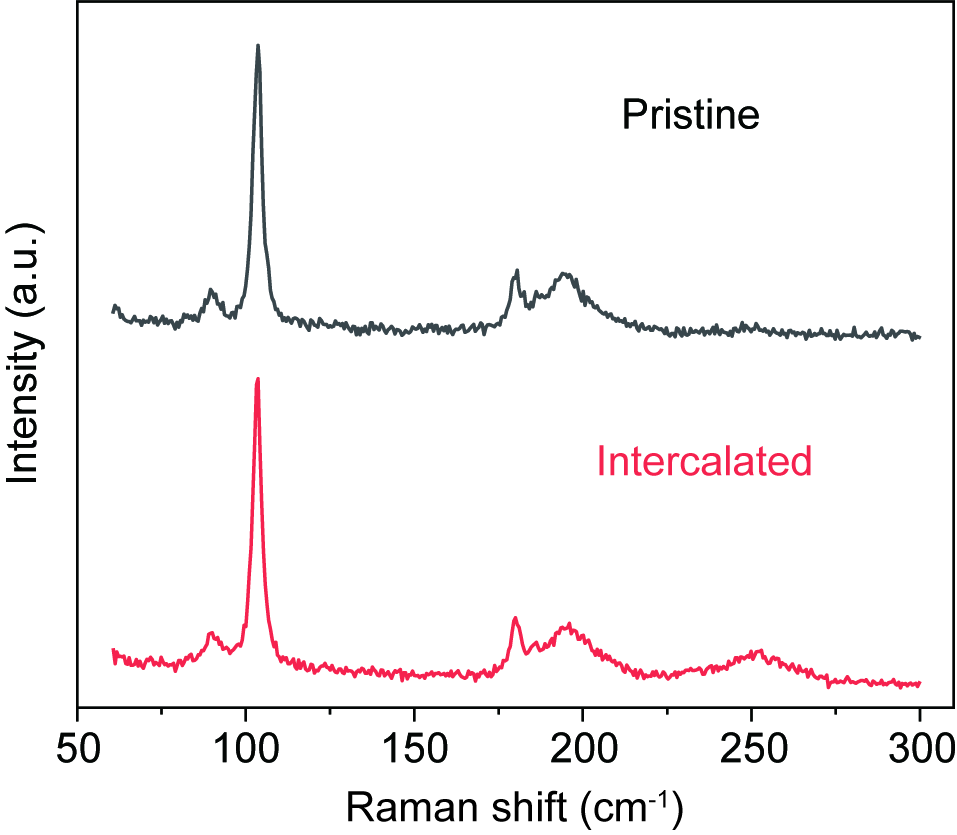


**Figure S5.** Raman spectra of another α-In_2_Se_3_ flake before and after Li-intercalation.
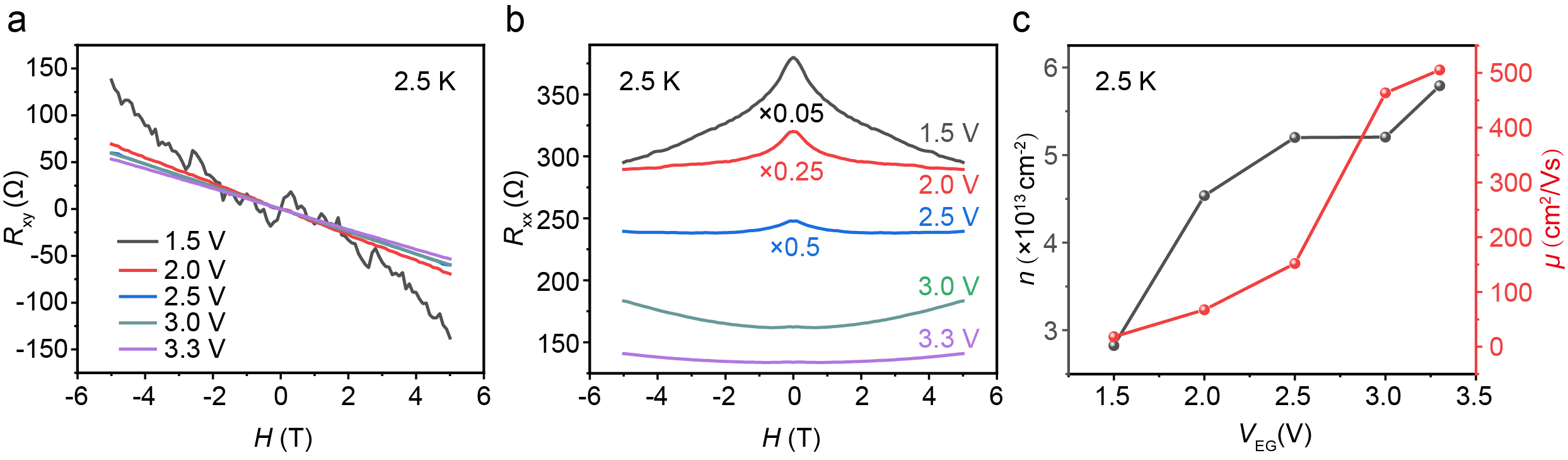
 **Figure S6.** Hall measurements for the ɑ-In_2_Se_3_Li_x_ device. a) Hall resistance of the ɑ-In_2_Se_3_Li_x_ device as a function of the magnetic field at various *V*_EG_, measured at 2.5 K. b) Magnetoresistance of the ɑ-In_2_Se_3_Li_x_ device as a function of the magnetic field at various gate voltages, measured at 2.5 K. c) *V*_EG_ dependent carrier density (*n*) and mobility (*μ*) at 2.5 K are calculated using $\sigma=ne\mu$ and $R_{H}={(ne)}^{-1}$ , where σ, e, and *R_H_* are the channel conductivity, the elementary charge, and the Hall coefficient, respectively.


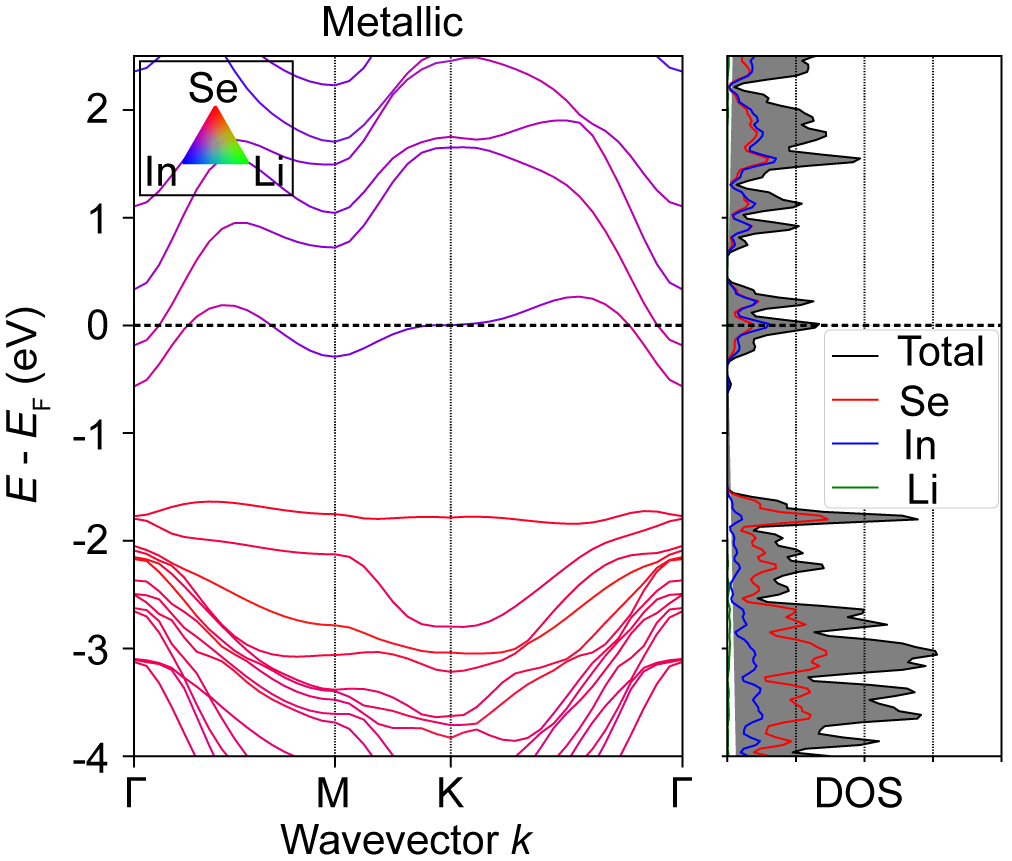


**Figure S7.** Calculated band structure for metallic bilayer ɑ-In_2_Se_3_ with intralayer lithium intercalation.


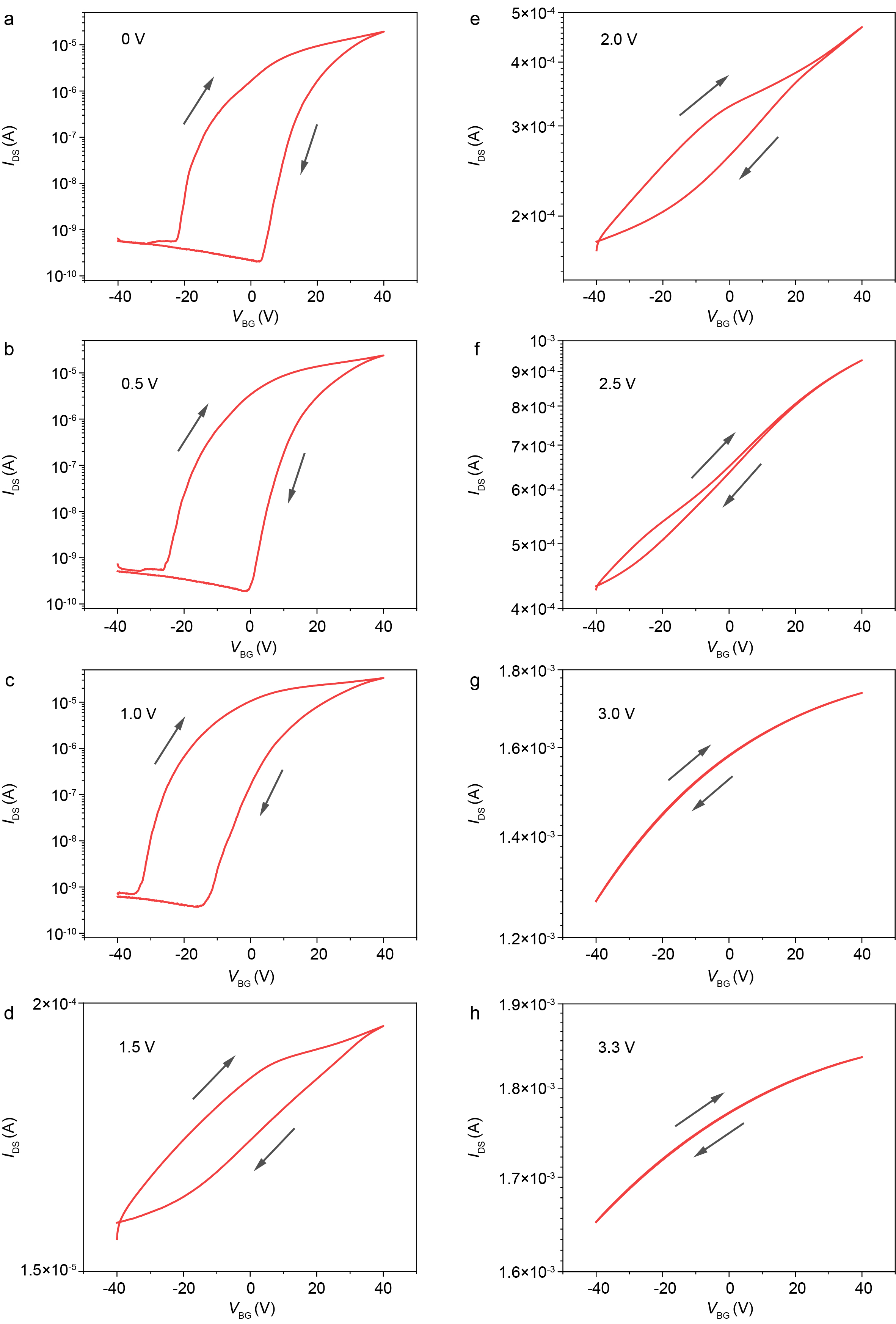


**Figure S8** Transfer characteristics. a-h) Evolution of the transfer curve of the ɑ-In_2_Se_3_Li_x_ device with increasing *V*_EG_ at 2.5 K. The drain-source voltage *V*_DS_ is 1 V.


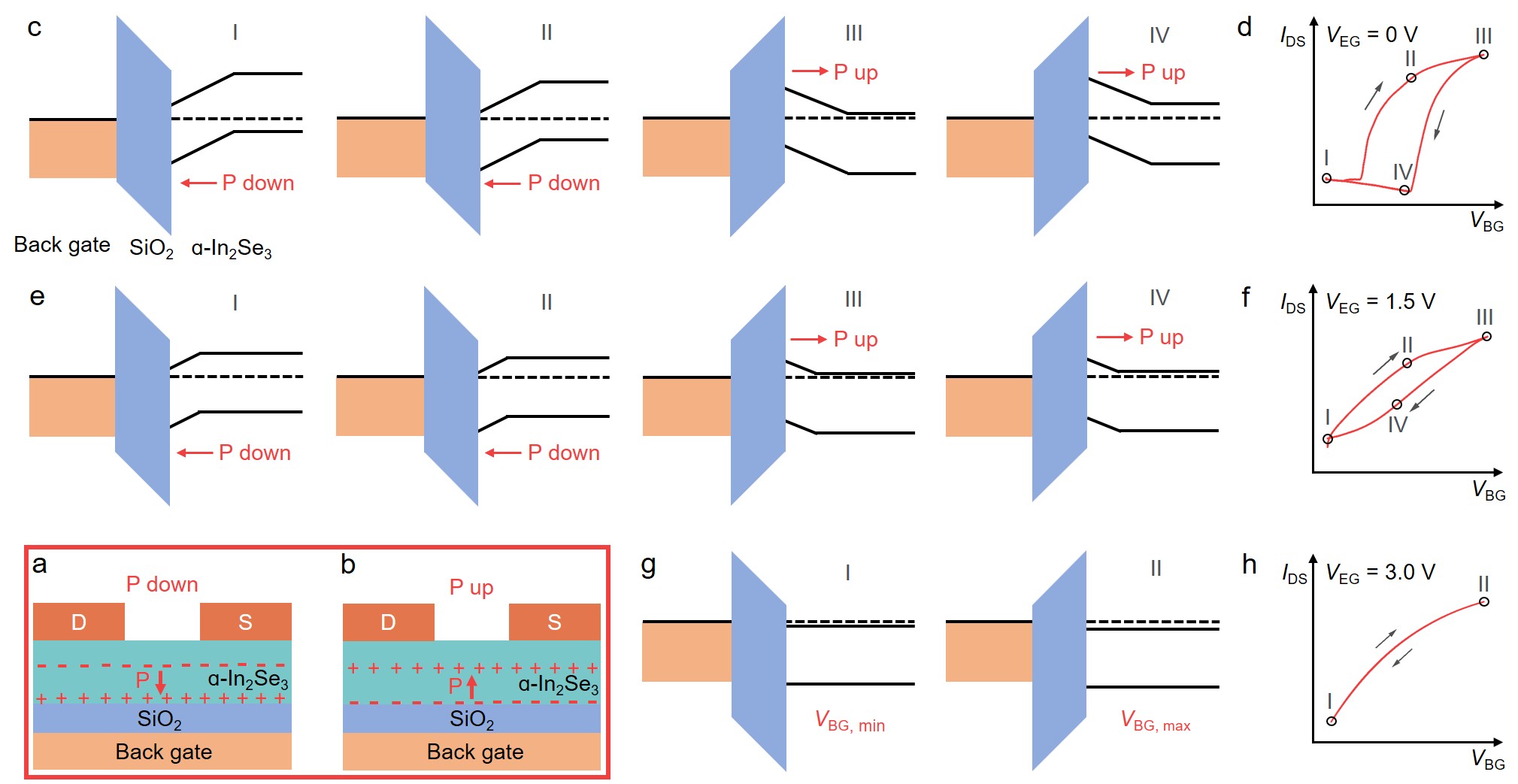


**Figure S9.** Schematic working mechanisms for an ɑ-In_2_Se_3_Li_x_ transistor under different lithium intercalation levels. a) Schematic of an ɑ-In_2_Se_3_Li_x_ transistor in the polarization (**P**) down state. b) Schematic of an ɑ-In_2_Se_3_Li_x_ transistor in the **P** up state. The plus and minus signs in the schematics represent the positive and negative bound charges, respectively. c) Band diagrams of the ɑ-In_2_Se_3_Li_x_ transistor slightly intercalated (*V*_EG_ = 0 V, or 0.5 V, or 1.0 V) at different states defined by the back gate. d) Representative transfer curve at *V*_EG_ = 0 V, in which the four different states (I, II, III, IV) in (c) are marked. e) Band diagrams of the ɑ-In_2_Se_3_Li_x_ transistor moderately intercalated (*V*_EG_ = 1.5 V, or 2.0 V, or 2.5 V) at different states defined by the back gate. f) Representative transfer curve at *V*_EG_ = 1.5 V, in which the four different states (I, II, III, IV) in (e) are marked. g) Band diagrams of the ɑ-In_2_Se_3_Li_x_ transistor enhancively intercalated (*V*_EG_ = 3.0 V or 3.3 V) at two different states, defined by the minimum (*V*_BG, min_) and maximum (*V*_BG, max_) *V*_BG_, respectively. h) Representative transfer curve at *V*_EG_ = 3.0 V, in which the two different states (I, II) in (g) are marked.

**Supplementary Note S1. Band diagrams of the α-In_2_Se_3_Li_x_ transistor at different lithium intercalation levels**

To reveal the physical mechanism behind the evolution of ferroelectricity with conductivity, we analyze how the band diagram of the α-In_2_Se_3_Li_x_ transistor varies with *V*_BG_ at different lithium intercalation levels. Figures S9a and S9b show the polarization bound change distribution in the polarization (**P**) down and up states, respectively. As the external electric field is not strong enough to penetrate the entire ɑ-In_2_Se_3_Li_x_ flake owing to the thick SiO_2_ layer (300 nm) and the screening effect of mobile charges, only a small portion of ferroelectric domains is reversed.

When the ɑ-In_2_Se_3_ flake is slightly intercalated (*V*_EG_ = 0, 0.5, or 1.0 V) and the minimum *V*_BG_ =−40 V is applied, the transistor is in the **P** down state and the energy band close to the bottom surface is bent downward owing to the bound charges (state I in Figure S9c). Because the Fermi level is far from the conduction band, the transistor is in an insulating state. When the gate voltage is increased until the Fermi level approaches the conduction band, *I*_DS_ suddenly increases and the transistor enters a conducting state (state II in Figure S9c). However, the applied *V*_BG_ is not large enough to reverse the polarization, so the transistor is still in the **P** down state. When the *V*_BG_ is further increased to the maximum value (40 V), the polarization is reversed and the transistor enters the **P** up state (state III in Figure S9c). Although the energy band is bent upward, the Fermi level is still close to the conduction band, so the transistor stays in the conducting state. When *V*_BG_ is decreased until the Fermi level is far from the conduction band, the transistor remains in the **P** up state yet becomes insulating again (state IV in Figure S9c). To understand the connection between the band diagram and the transport property, these four states above are marked in the representative transfer curve at *V*_EG_ = 0 V in Figure S9d. Moreover, the forward and backward sweeps of *V*_BG_ correspond to different band diagrams, i.e., those bending downward and upward, respectively. Hence, the conductivity of the ɑ-In_2_Se_3_Li_x_ channel differs in the two sweep directions of *V*_BG_ and a hysteresis loop appears in the transfer curve. If the electric field (i.e., *V*_BG_) is increased, the polarization of more domains would be switched. As a result, the energy bands of more areas would bend and thus the hysteresis loop would grow larger (Figure S4).

When the ɑ-In_2_Se_3_ flake is moderately intercalated (*V*_EG_ = 1.5, 2.0, or 2.5 V), a similar evolution of the band diagram with *V*_BG_ can be observed (Figure S9e) and the four states are also marked in the representative transfer curve at *V*_EG_ = 1.5 V in Figure S9f. Notably, compared with the slight intercalation scenario, there are two distinct features in the moderate intercalation scenario. 1) The electron density increases considerably owing to the increased Li ions intercalated, so the Fermi level is closer to the bottom of the conduction band. 2) Owing to the large quantity of injected electrons, the screening effect on the external electric field and the perturbation on long-range Coulomb forces are enhanced. Thus, the penetration depth of the electric field is shortened and the ferroelectricity is weakened. Therefore, the area with a bent energy band shrinks, as does the hysteresis loop.

When the ɑ-In_2_Se_3_ flake is enhancively intercalated (*V*_EG_ = 3.0 or 3.3 V), more electrons flow into the ɑ-In_2_Se_3_Li_x_ channel and the external electric field is completely screened. Therefore, no energy band is bent, and the ferroelectric hysteresis loop disappears (Figure S9g). The Fermi level shows only a slight shift in the conduction band when *V*_BG_ is swept from *V*_BG, min_ (−40 V) to *V*_BG, max_ (40 V) owing to the high carrier density. These two states are also marked in the representative transfer curve at *V*_EG_ = 3.0 V in Figure S9h.
